# Intraoperative Fluoroscopy Allows the Reliable Assessment of Deformity Correction during Periacetabular Osteotomy

**DOI:** 10.3390/jcm11164817

**Published:** 2022-08-17

**Authors:** Johannes Christian Reichert, André Hofer, Georg Matziolis, Georgi Iwan Wassilew

**Affiliations:** 1 Center for Orthopaedics, Trauma Surgery and Rehabilitation Medicine, University Medicine Greifswald, Ferdinand-Sauerbruch-Straße, 17475 Greifswald, Germany; 2 Orthopaedic Department, Campus Eisenberg, University Hospital Jena, Klosterlausnitzer Str. 81, 07607 Eisenberg, Germany

**Keywords:** periacetabular osteotomy, fluoroscopy, hip dysplasia, wall index

## Abstract

We aimed to determine the accuracy and reliability of measures characterizing anterior, lateral, and posterior acetabular coverage on intraoperative fluoroscopic images compared to postoperative radiographs when performing periacetabular osteotomies (PAOs). A study involving 100 PAOs was initiated applying a standardized intraoperative imaging protocol. Coverage was determined by the lateral center edge angle (LCEA), the Tönnis angle (TA), and the anterior and posterior wall index (AWI, PWI). An intraclass correlation coefficient (ICC) model was used to assess interrater (ICC (3,2)) and intrarater (ICC (2,1)) reliability. The ICC (2,2) between analyses obtained from intraoperative fluoroscopy and postoperative radiographs and the corresponding 95% confidence interval (CI) were determined and complemented by Bland–Altman analysis, the mean difference, and 95% limits of agreement (LOA). The ICCs were 0.849 for the LCEA (95% CI 0.783–0.896), 0.897 for the TA (95% CI 0.851–0.930), 0.864 for the AWI (95% CI 0.804–0.907), and 0.804 for the PWI (0.722–0.864). The assessed interrater reliability was excellent except for the AWI, which was graded good (ICC = 0.857, 95% CI 0.794–0.902). Interrater agreement was generally good and fair for the AWI (ICC = 0.715, 95% CI 0.603–0.780). For each postoperative radiograph, interrater reliability was good with ICCs ranging from 0.813 (TA) to 0.881 (PWI). Intrarater reliability was good for all measurements and excellent for the preoperative TA (ICC = 0.993, 95% CI 0.984–0.997) and PWI (ICC = 0.954, 95% CI 0.919–0.97). In summary, we confirm the validity and reliability of intraoperative fluoroscopy as an alternative imaging modality to radiography to evaluate acetabular fragment orientation during PAO. We affirm the LCEA and TA as precise measures for lateral head coverage, and show the suitability of the AWI and PWI to steadily assess acetabular version.

## 1. Introduction

Over the years, periacetabular osteotomy (PAO) has become increasingly popular. At present, PAO represents the preferable treatment option for symptomatic, skeletally mature patients suffering from acetabular dysplasia [1]. Congruent articular surfaces and an intact cartilage with an osteoarthritis grade ≤1 (Tönnis), however, are a premise [2]. The complex three-dimensional reorientation of the acetabular segment to optimize femoral head coverage remains the decisive step determining the long-term outcome [3]. Consequently, the intraoperative evaluation of deformity correction by radiography, fluoroscopy, or computer-assisted marker tracking is essential. Most commonly, surgeons rely on fluoroscopic control of the performed osteotomies and fragment realignment. This reduces exposition to ionizing radiation [4]. However, fluoroscopy provides a posteroanterior (PA) view of the hip, while radiography provides an anteroposterior (AP) image. Therefore, it remains unclear to what extent the inherent difference in projection affects the assessment of acetabular version by the anterior (AWI) and posterior wall indices (PWI) [5].

Previous studies have demonstrated a reliable intraoperative fluoroscopic judgment of lateral coverage and the anterior center edge angle (ACEA) [6,7,8]. However, these either included rather small numbers of patients, only single evaluators, or did not consider acetabular version. Reports that do include version assessment lack standardized investigational protocols, compare supine with standing projections, and are subject to selection bias [9]. 

It is worth emphasizing that standing results in a backward tilt of the pelvis, an effect that appears more pronounced in women [10]. Furthermore, standing affects acetabular version [10,11] and significantly influences measurements of the LCEA [12].

To address these shortcomings, we initiated a study involving 100 PAOs performed by a single surgeon applying a standardized intraoperative imaging protocol. We aimed to determine the accuracy and reliability of measures characterizing anterior, lateral, and posterior acetabular coverage based on fluoroscopic images when compared to postoperative radiographs.

## 2. Materials and Methods

Between January 2019 and April 2021, 190 consecutive PAOs were performed by the corresponding author. Patients enrolled were selected from our institutional database. All patients were symptomatic and had congruent articular surfaces presenting no signs of progressed osteoarthritis (Tönnis grade ≤ 1). The PAOs were performed using a rectus-sparing approach as described previously [13]. Patients were included if they had preoperative and postoperative supine AP pelvic radiographs, and fluoroscopy images including final images with hardware in place in our picture archiving and communication system (PACS). Fluoroscopic images were acquired using a Philips Veradius Unity C-arm. For 90 patients, the intraoperative images were not digitally available in the PACS but archived in a conventional analogue fashion. Consequently, we were able to analyze the images of 100 patients. Demographics of the patients enrolled were as follows: Their mean age at surgery was 30.8 years (15–50 years). Sixty percent of the hips were right sided. Seventy-three percent of the patients were female. All patients had a body mass index (BMI) < 30 kg/m^2^. None of the cases had previously undergone unilateral or contralateral interventions. The preoperative supine AP radiographs of the pelvis were used to measure the lateral center edge angle (LCEA), the Tönnis angle (TA), and the anterior and posterior wall index (AWI, PWI), as described previously [5,14]. The measurements were taken utilizing the TraumaCad software (Brainlab, Munich, Germany). Two qualified observers performed all measurements. They were blinded to each other and between imaging techniques, respectively. One observer repeated the analyses on a random sample of 50 hips blinded to the previous results to assess intrarater reliability, with a minimum of eight weeks between measurements. 

**Figure 1:**
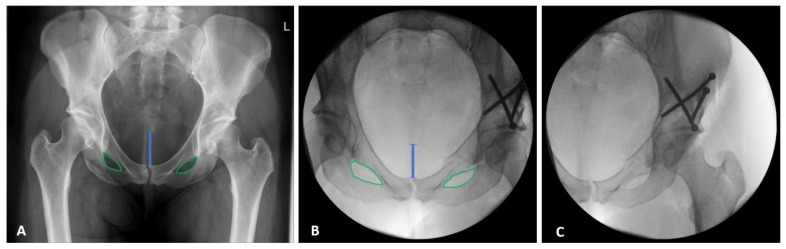
Preoperative radiograph (**A**) and intraoperative fluoroscopy (**B**) of the pelvic ring and the obturator foramen (green line) with adjusted tilt, rotation, and coccyx alignment, and with the distance (blue line) from the symphysis to match the preoperative radiograph. Visualized operated hip after lateralization of the C-arm for image acquisition (**C**).

A standardized approach was used to obtain the fluoroscopic images during surgery: The C-arm fluoroscopy machine was positioned to acquire an image of the pelvic ring and the obturator foramen. Consecutively, tilt and rotation were adjusted to match the preoperative pelvic radiograph. Care was taken to closely reproduce the coccyx alignment with the pubic symphysis and the distance from the coccyx to the symphysis, as well as the shape and symmetry of the obturator foramina. After fulfillment of these criteria, the C-arm was moved to visualize the operated hip for image acquisition (Figure 1).

Statistical analysis was performed using SPSS 19.0 (IBM, Armonk, New York, NY, USA). An intraclass correlation coefficient (ICC) model was used to assess interrater (ICC (3,2)) and intrarater (ICC (2,1)) reliability [15]. The interpretation of the results followed recommendations by Koo and Li [16]. An ICC < 0.5 was considered poor, between 0.5 and 0.75 fair, between 0.75 and 0.9 good, and >0.9 excellent.

Furthermore, the ICC (2,2) between analyses obtained from intraoperative fluoroscopy and postoperative radiographs, as well as the corresponding 95% confidence interval (CI), was determined. ICC estimates were complemented by Bland–Altman analysis and calculation of the mean difference and 95% limits of agreement (LOA) [17].

## 3. Results

In general, measurements on fluoroscopic images and postoperative radiographs show good conformity. Consequently, the intraclass correlation coefficients for the LCEA, TA, AWI, and PWI showed a good match between intraoperative fluoroscopy and postoperative radiographs. The ICCs were 0.849 for the LCEA (95% CI 0.783–0.896), 0.897 for the TA (95% CI 0.851–0.930), 0.864 for the AWI (95% CI 0.804–0.907), and 0.804 for the PWI (0.722–0.864) (Table 1). The conducted Bland–Altman analysis precluded the relevant effects of systematic bias when comparing the respective modes of image acquisition (mean differences: LCEA 0.56°, TA 0.3°, AWI 0.007, PWI 0.010) (Table 1). The reorientation of the acetabular segment resulted in a mean correction of 10° for the LCEA, 8.1° for the TA, 0.01 for the AWI, and 0.08 for the PWI (Table 2). 

The assessed interrater reliability regarding the preoperative radiographic measurements was excellent except for the AWI, which was graded as good (ICC = 0.857, 95% CI 0.794–0.902). The evaluation of the intraoperative images showed good interrater agreement aside from the anterior wall index, which was ranked as fair (ICC = 0.715, 95% CI 0.603–0.780) (Table 3). For the values obtained from postoperative radiographs, interrater reliability was good in each case, with ICCs ranging from 0.813 (TA) to 0.881 (PWI) (Table 3).

Intrarater reliability was rated good for all measurements and even excellent for the preoperative TA (ICC = 0.993, 95% CI 0.984–0.997) and PWI (ICC = 0.954, 95% CI 0.919–0.974), as well as the intraoperative LCEA (ICC = 0.960, 95% CI 0.930–0.977) (Table 3).

## 4. Discussion

Developmental dysplasia of the hip is a leading cause of secondary osteoarthritis. The main morphological characteristics include an insufficient coverage of the femoral head and a disproportionately shallow acetabulum in association with labral hypertrophy. The general incidence varies between 3 and 5% [18]. Untreated dysplastic hips result in considerable pain and impaired joint function, and favor the development of osteoarthritis. Consequently, the surgical correction and improvement of femoral head coverage is desirable in young adults with unharmed cartilage.

When performing PAO, the surgeon commonly relies on visual cues and lacks a reliable method to precisely quantify the level of correction [19]. These visual cues are usually obtained by fluoroscopy since intraoperative radiography is time-consuming, is associated with a higher radiation dose, and relies on infrastructural preconditions. Fluoroscopy, however, is easily available but generates a posteroanterior view of the hip with a limited field of view and resolution, thus differing from anteroposterior radiographic images. It is known that AP and PA projections can lead to changed radiographic appearances influencing image interpretation [20]. Compared to AP projections, fluoroscopic PA images result in a decreased distance between the pubic symphysis and sacrococcygeal junction. Consecutively, the acetabulum imposes with more anteversion [4].

In the current study, we therefore compared images of 100 cases in supine position to be able to determine the effect of AP and PA projection on the assessment of acetabular inclination and version represented by the anterior (AWI) and posterior wall indices (PWI) [5]. The present outcome analysis indicates that the AWI and PWI, which represent a means to estimate anterior and posterior coverage, are reliable. The values measured intraoperatively correlated well with those determined on postoperative radiographs, respectively (ICC 0.864 and 0.804). This is in contrast to the statements of Wylie et al., who determined a considerably lower correlation (ICC 0.63 and 0.72) for intra- and postoperative AWI and PWI measurements [9]. One reason might be that in our study, postoperative control radiographs were taken in a supine position. This results in an average change in the pelvic tilt of about 7 degrees in males and 14 degrees in females [21], also affecting the assessment of acetabular version [10]. Having reliable indices to comprehensively assess version helps to avoid retroversion and deviations of the anterior wall index, which are known to result in inferior postoperative outcomes [22], and to decrease the survival of native hip joints after PAO [23]. Therefore, besides meeting other well-recognized radiographic recommendations, the acetabular wall indices can eventually be determined intraoperatively to ascertain the desired adequate reorientation of the acetabular segment, with the aim to reduce the risk of a future conversion to total hip arthroplasty.

Our study results confirm previous reports that the determination of the LCEA and TA allow for an accurate assessment of the lateral acetabular coverage [6,7,8], since the measurements taken on intraoperative fluoroscopic images and postoperative radiographs showed good agreement (ICC 0.849 and 0.897). Consequently, LCEA and TA measurements can help to circumvent both acetabular undercoverage and overcoverage, which are known to drive the development of degenerative changes in the hip joint, ultimately resulting in osteoarthritis. Undercoverage increases joint contact forces [24] and leads to static overload [25]. Acetabular overcoverage, on the other hand, causes a mechanical conflict between the acetabular rim and the femoral head–neck junction [26], damaging labral structures [27]. It must be pointed out that the above-mentioned change in pelvic tilt during standing also influences measurements of the LCEA [12].

In summary, our data confirm the validity and reliability of intraoperative fluoroscopy as an alternative imaging modality to radiography to evaluate acetabular fragment orientation when performing PAOs. We affirm the LCEA and TA as precise measures for lateral head coverage, and show the suitability of the AWI and PWI to steadily assess acetabular version.

## Figures and Tables

**Table 1:** Agreement between intraoperative fluoroscopy and postoperative radiograph (*n* = 100).

Acetabular Measurement	ICC	95% CI	Mean Difference	Standard Deviation (+/−)	95% Limits of Agreement
LCEA	0.849	0.783–0.896	−0.560	1.351	−3.208–2.088
TA	0.897	0.851–0.930	0.300	0.980	−1.620–2.220
AWI	0.864	0.804–0.907	−0.007	0.057	−0.118–0.105
PWI	0.804	0.722–0.864	0.010	0.110	−0.205–0.226

ICC: intraclass correlation coefficient, CI: confidence interval, LCEA: lateral center-edge angle, TA: Tönnis angle, AWI: anterior wall index, PWI: posterior wall index.

**Table 2:** Summary of acetabular measurements (*n* = 100).

	Pre		Intra		Post		Correction	
Acetabular Measurement	Mean	SD	Mean	SD	Mean	SD	Mean	95% CI
LCEA	20.6	7.46	30.1	3.65	30.7	3.95	10.1	8.2–10.8
TA	12.2	6.16	4.4	2.84	4.1	2.67	−8.1	7.1–9.1
AWI	0.38	0.13	0.36	0.11	0.37	0.11	−0.01	−0.04–0.01
PWI	0.81	0.15	0.90	0.19	0.89	0.16	0.08	0.05–0.10

ICC: intraclass correlation coefficient, CI: confidence interval, LCEA: lateral center-edge angle, TA: Tönnis angle, AWI: anterior wall index, PWI: posterior wall index.

**Table 3:** Interrater and intrarater reliability.

Acetabular Measurement	Interrater Reliability *n* = 100		Intrarater Reliability *n* = 50	
**Preoperative**	**ICC**	**95% CI**	**ICC**	**95% CI**
LCEA	0.965	0.938–0.980	0.887	0.753–0.950
TA	0.914	0.875–0.941	0.993	0.984–0.997
AWI	0.857	0.794–0.902	0.813	0.691–0.890
PWI	0.926	0.892–0.950	0.954	0.919–0.974
**Intraoperative**				
LCEA	0.861	0.800–0.905	0.960	0.930–0.977
TA	0.849	0.783–0.896	0.857	0.760–0.917
AWI	0.715	0.603–0.780	0.762	0.614–0.858
PWI	0.792	0.705–0.855	0.845	0.741–0.910
**Postoperative**				
LCEA	0.834	0.763–0.885	0.886	0.810–0.934
TA	0.813	0.734–0.870	0.872	0.784–0.926
AWI	0.844	0.776–0.893	0.801	0.673–0.883
PWI	0.881	0.828–0.919	0.869	0.779–0.924

## Data Availability

The data that support the findings of this study are available on request from the corresponding author. The data are not publicly available due to privacy or ethical restrictions.

## References

[1] Wyles C.C., Vargas J.S., Heidenreich M.J., Mara K.C., Peters C.L., Clohisy J.C., Trousdale R.T., Sierra R.J. (2020). Hitting the Target: Natural History of the Hip Based on Achieving an Acetabular Safe Zone Following Periacetabular Osteotomy. J. Bone Jt. Surg..

[2] Millis M.B., McClincy M. (2018). Periacetabular osteotomy to treat residual dysplasia in adolescents and young adults: Indications, complications, results. J. Child. Orthop..

[3] Lerch T.D., Steppacher S.D., Liechti E.F., Tannast M., Siebenrock K.A. (2017). One-third of Hips After Periacetabular Osteotomy Survive 30 Years with Good Clinical Results, No Progression of Arthritis, or Conversion to THA. Clin. Orthop. Relat. Res..

[4] Kosuge D., Cordier T., Solomon L.B., Howie D.W. (2014). Dilemmas in imaging for peri-acetabular osteotomy: The influence of patient position and imaging technique on the radiological features of hip dysplasia. Bone Jt. J..

[5] Siebenrock K.A., Kistler L., Schwab J., Büchler L., Tannast M. (2012). The Acetabular Wall Index for Assessing Anteroposterior Femoral Head Coverage in Symptomatic Patients. Clin. Orthop. Relat. Res..

[6] Kühnel S.P., Kalberer F.A., Dora C.F. (2011). Periacetabular osteotomy: Validation of intraoperative fluoroscopic monitoring of acetabular orientation. Hip Int..

[7] Lehmann C.L., Nepple J.J., Baca G., Schoenecker P.L., Clohisy J.C. (2012). Do fluoroscopy and postoperative radiographs correlate for periacetabular osteotomy corrections?. Clin. Orthop. Relat. Res..

[8] Wylie J., Ross J.A., Erickson J.A., Anderson M.B., Peters C.L. (2016). Operative Fluoroscopic Correction Is Reliable and Correlates With Postoperative Radiographic Correction in Periacetabular Osteotomy. Clin. Orthop. Relat. Res..

[9] Wylie J.D., Ferrer M.G., McClincy M.P., Miller P.E., Millis M.B., Kim Y.-J., Novais E.N. (2019). What Is the Reliability and Accuracy of Intraoperative Fluoroscopy in Evaluating Anterior, Lateral, and Posterior Coverage During Periacetabular Osteotomy?. Clin. Orthop. Relat. Res..

[10] Troelsen A., Jacobsen S., Rømer L., Søballe K. (2008). Weightbearing Anteroposterior Pelvic Radiographs are Recommended in DDH Assessment. Clin. Orthop. Relat. Res..

[11] Siebenrock K.A., Kalbermatten D.F., Ganz R. (2003). Effect of Pelvic Tilt on Acetabular Retroversion: A Study of Pelves From Cadavers. Clin. Orthop. Relat. Res..

[12] Fuchs-Winkelmann S., Peterlein C.-D., Tibesku C.O., Weinstein S.L. (2008). Comparison of Pelvic Radiographs in Weightbearing and Supine Positions. Clin. Orthop. Relat. Res..

[13] Khan O.H., Malviya A., Subramanian P., Agolley D., Witt J.D. (2017). Minimally invasive periacetabular osteotomy using a modified Smith-Petersen approach: Technique and early outcomes. Bone Jt. J..

[14] McClincy M.P., Wylie J.D., Yen Y.M., Novais E.N. (2019). Mild or Borderline Hip Dysplasia: Are We Characterizing Hips With a Lateral Center-Edge Angle Between 18 degrees and 25 degrees Appropriately?. Am. J. Sports Med..

[15] Shrout P.E., Fleiss J.L. (1979). Intraclass correlations: Uses in assessing rater reliability. Psychol. Bull..

[16] Koo T.K., Li M.Y. (2016). A Guideline of Selecting and Reporting Intraclass Correlation Coefficients for Reliability Research. J. Chiropr. Med..

[17] Bland J.M., Altman D.G. (1986). Statistical methods for assessing agreement between two methods of clinical measurement. Lancet.

[18] Ortiz-Neira C.L., Paolucci E.O., Donnon T. (2012). A meta-analysis of common risk factors associated with the diagnosis of developmental dysplasia of the hip in newborns. Eur. J. Radiol..

[19] Hooper J.M., Mays R.R., Poultsides L.A., Castaneda P.G., Muir J.M., Kamath A.F. (2019). Periacetabular osteotomy using an imageless computer-assisted navigation system: A new surgical technique. J. Hip Preserv. Surg..

[20] Lai V., Tsang W.K., Chan W.C., Yeung T.W. (2012). Diagnostic accuracy of mediastinal width measurement on posteroanterior and anteroposterior chest radiographs in the depiction of acute nontraumatic thoracic aortic dissection. Emerg. Radiol..

[21] Tannast M., Murphy S.B., Langlotz F., Anderson S.E., Siebenrock K.A. (2005). Estimation of pelvic tilt on anteroposterior X-rays—a comparison of six parameters. Skelet. Radiol..

[22] Goronzy J., Franken L., Hartmann A., Thielemann F., Blum S., Günther K.-P., Nowotny J., Postler A. (2020). Acetabular- and femoral orientation after periacetabular osteotomy as a predictor for outcome and osteoarthritis. BMC Musculoskelet. Disord..

[23] Stetzelberger V.M., Leibold C.S., Steppacher S.D., Schwab J.M., Siebenrock K.A., Tannast M. (2021). The Acetabular Wall Index Is Associated with Long-term Conversion to THA after PAO. Clin. Orthop. Relat. Res..

[24] Hipp J., Sugano N., Millis M.B., Murphy S.B. (1999). Planning Acetabular Redirection Osteotomies Based on Joint Contact Pressures. Clin. Orthop. Relat. Res..

[25] Jacobsen S., Sonne-Holm S., Soballe K., Gebuhr P., Lund B. (2005). Hip dysplasia and osteoarthrosis: A survey of 4151 subjects from the Osteoarthrosis Substudy of the Copenhagen City Heart Study. Acta Orthop..

[26] Ganz R., Parvizi J., Beck M., Leunig M., Nötzli H., Siebenrock K.A. (2003). Femoroacetabular impingement: A cause for osteoarthritis of the hip. Clin. Orthop. Relat. Res..

[27] Tannast M., Goricki D., Beck M., Murphy S.B., Siebenrock K.A. (2008). Hip Damage Occurs at the Zone of Femoroacetabular Impingement. Clin. Orthop. Relat. Res..

